# Effect of combined inhibition of p110 alpha PI3K isoform and STAT3 pathway in ovarian cancer platinum-based resistance

**DOI:** 10.18632/oncotarget.25513

**Published:** 2018-06-05

**Authors:** Augustin Le Naour, Renaud Mevel, Benoit Thibault, Elise Courtais, Elodie Chantalat, Jean Pierre Delord, Bettina Couderc, Julie Guillermet-Guibert, Alejandra Martinez

**Affiliations:** ^1^ Centre de Recherches en Cancérologie de Toulouse (CRCT), UMR 1037 INSERM, University Toulouse III, Toulouse, France; ^2^ Department Surgical Oncology, Institut Claudius Regaud, Institut Universitaire du Cancer Toulouse-Oncopole, Toulouse, France; ^3^ Department Medical Oncology, Institut Claudius Regaud, Institut Universitaire du Cancer Toulouse-Oncopole, Toulouse, France; ^4^ Department Biology, Institut Claudius Regaud, Institut Universitaire du Cancer, Toulouse, France; ^5^ Laboratoire d'excellence LABEX TouCAN, Toulouse, France

**Keywords:** ovarian cancer, IL-6, chemoresistance, PI3KCA, ascites

## Abstract

**Background:**

Ovarian cancer is associated with poor prognostic outcome due to late diagnosis and to intrinsic and acquired resistance to platinum-based chemotherapy in a large number of patients. This chemoresistance is acquired through the peritoneal and ascites microenvironment by several released factors, such as IL-6,. Preclinical studies have implicated the activation of PI3K pathway in chemoresistance, showing it to extend tumor cell survival and modulate multidrug resistance. We aimed to evaluate the implication of the p110 alpha PI3K subunit in ovarian cancer chemoresistance acquisition, and to evaluate whether the STAT3 pathway can mediate resistance to PI3K inhibitors through secretion of IL6.

**Results:**

Human ovarian adenocarcinoma IGROV-1 and JHOC-5 cells cultured in ascites showed an increase in carboplatinum-based resistance. Level of chemoresistance was associated to IL6 concentration in ascites. Activation of PI3K/Akt, STAT and MAPK pathways was observed after IGROV-1 incubation with ascites and treatment with carboplatin. Neither IGROV-1 nor JHOC-5 cells exposed to ascites treated with additional IL-6 directed antibody showed any reversion of the chemoresistance.

**Conclusion:**

IL6-related resistance was not abolished by the selective inhibition of PI3K alpha subunit coupled with the anti-IL6-receptor antibody tocilizumab. This dual inhibition requires further exploration in other ovarian cancer models such as clear cell carcinoma.

## INTRODUCTION

The 5-year overall survival for advanced ovarian cancer is approximately 30% [1]. This low survival rate is due to the diagnosis of ovarian cancer at advanced stages of disease and to the intrinsic and acquired resistance to platinum-based chemotherapy in a large number of patients [2]. Epithelial ovarian cancer can be divided into five different diseases according to their pathology: low-grade and high-grade serous carcinomas, then mucinous, endometrioid and clear cell carcinomas [3]. Each of these subtypes is genetically distinct with different underlying molecular abnormalities and different morphological, immunohistochemical and clinical characteristics [4, 5]. Prognostic factors including patient’s age, performance status, FIGO stage, histological tumor grade and subtype, and initial surgery results, are insufficient to account for large differences in response to chemotherapy and survival among patients. Indeed, different molecular profiles and different prognostic outcome can be found for any particular histological type [6, 7]. While high grade serous adenocarcinoma is associated with initial sensitivity to chemotherapy, some rare histological subtypes, such as clear cell carcinoma, are resistant to conventional platinum-based chemotherapy [8].

Regarding patients with chemosensitive ovarian carcinomas, approximately 70% of them will initially respond to a combination of platinum and taxane-based chemotherapy but over half will then recur with a frequent acquisition of resistance to platins [9]. Indeed, small numbers of drug-resistant cells can persist for many months and remain dormant in the peritoneal cavity, leading to peritoneal recurrent disease. This unique metastatic pattern suggests a strong tropism of ovarian carcinoma for the peritoneal cavity which most probably displays a specifically permissive microenvironment for tumor growth through the release of cytokines and growth factors able to activate specific signaling pathways in tumor cells [10]. In a previous study, we compared the transcriptomic profiles of primary ovarian tumors and their matched peritoneal metastases. We found that peritoneal lesions overexpressed genes encoding proteins involved in ‘cytokine-cytokine receptor interaction’ and in the ‘Jak-STAT signaling pathway’ [11–13]. Among them, IL-6 is one of the major immunoregulatory cytokines with a role in tumor proliferation, invasion, angiogenesis and chemoresistance. It has been implicated in chemoresistance acquisition in several malignancies via several mechanisms [11–13]. Concerning ovarian cancers, we have shown that IL-6 could be overexpressed by the ovarian adenocarcinoma cells themselves or by cells within their microenvironment [14, 15]. Moreover, high serum levels of proinflammatory cytokines such as IL-6 (or IL-8) were noted in the ascites of epithelial ovarian cancer patients presenting with a poor prognosis [16, 17]. Finally, several authors have reported that IL-6 secretion can lead to resistance of ovarian tumor cells to treatment by carboplatin and paclitaxel [18, 19]. We have previously reported copy number differences between primary and peritoneal implants in genes mainly implicated in the kinase activation network [20]. Interestingly, despite the large number of genes in this global network, only a small number (22 genes) were found to be implicated in kinase activation within early peritoneal implants, mostly involving the PI3K pathway [20]. We confirmed these observations in another study in which we performed a combined genomic and transcriptomic analysis on multi-site samples from ovarian cancer patients and identified preferentially-expressed alleles that could act as cancer drivers and thus be used as therapeutic targets [21]. One of the genes showing significant allele fraction differences was *PIK3CA* [21], which encodes the catalytic subunit of PI3Kα isoform (p110α). Class I PI3Ks are heterodimeric proteins constituted by a catalytic subunit (4 isoforms with non redundant activities: p110α, p110β, p110δ and p110γ) and one or several regulatory subunits [22–24]. Somatic mutations with gain-of-function in the *PIK3CA* gene are found in 12% of high-grade serous ovarian carcinomas. One preclinical study implicated the activation of the PI3K pathway in resistance to chemotherapy via an extended cell survival and modulation of multidrug resistance [25]. Blockade of the PI3K pathway sensitized tumor cells to platinum and taxanes, and combinatorial treatment with chemotherapy resulted in increased antitumor activity in multiple human xenograft models of breast, lung cancer, and glioblastoma grown in nude mice.

On the basis of these findings, several clinical trials are currently examining PI3K/Akt/mTORC1 axis inhibition [26]. These trials involve pan-inhibitors of the PI3K pathway and target the four isoforms of class I PI3K, usually also inhibiting the downstream effector mTOR. Unfortunately, these inhibitors display adverse effects at high doses and at lower doses are not as efficient as expected, likely due to the activation of compensatory pathways or to the insufficient drug dosage needed to reach the targets.

We have previously demonstrated the specific implication of the p110α isoform of Class I PI3Ks in tumor initiation or progression in pancreatic cancers [27, 28]. The development of isoform-selective PI3K inhibitors that would enable a complete blockade of the relevant target with limited toxicity compared with pan-PI3K inhibitors is of major current interest, in particular for future combination therapies. Regarding the potential implication of the different isoforms of PI3K within the various stromal cells involved in tumor progression, Niedermeier *et al.* identified that p110α-specific inhibitors could counteract the chemoresistance shown by chronic lymphoid leukemia, by suppressing the protective effect of marrow stromal cells on fludarabine-induced apoptosis [29]. However, the precise mechanism of resistance to PI3K/Akt/mTOR inhibitors remains unclear. Genetic and pharmacological data from our laboratory demonstrate that PI3K signaling in other cancers where the stromal compartment plays a major role regulates the autocrine IL-6-STAT3 loop [27]. In breast cancer, this IL-6-STAT3 positive feedback loop was shown to mediate resistance to PI3K inhibitors through an epithelial-mesenchymal transition of breast cancer cells and expansion of cancer stem cells. Overlapping increase in IL-6 production was associated with a significant increase in STAT3 activity and PI3K inhibitor resistance. Treatment combination using STAT3 and PI3K inhibitors suppressed both STAT3 and Akt activities and induced the cleavage of caspase 3, a well-known apoptotic marker [30]. These results in breast cancer are promising with regards to dual inhibition of IL-6 and PI3K potentially broadening ovarian cancer treatment possibilities.

The aim of our present study was to evaluate the implication of the p110α PI3K subunit in ovarian cancer chemoresistance acquisition, and to evaluate whether the JAK/STAT pathway could mediate resistance to PI3K inhibitors through secretion of IL-6.

## RESULTS

### Ascites medium confers chemoresistance to human ovarian cancer cells (HOCCs) through secreted factors

**Figure 1:**
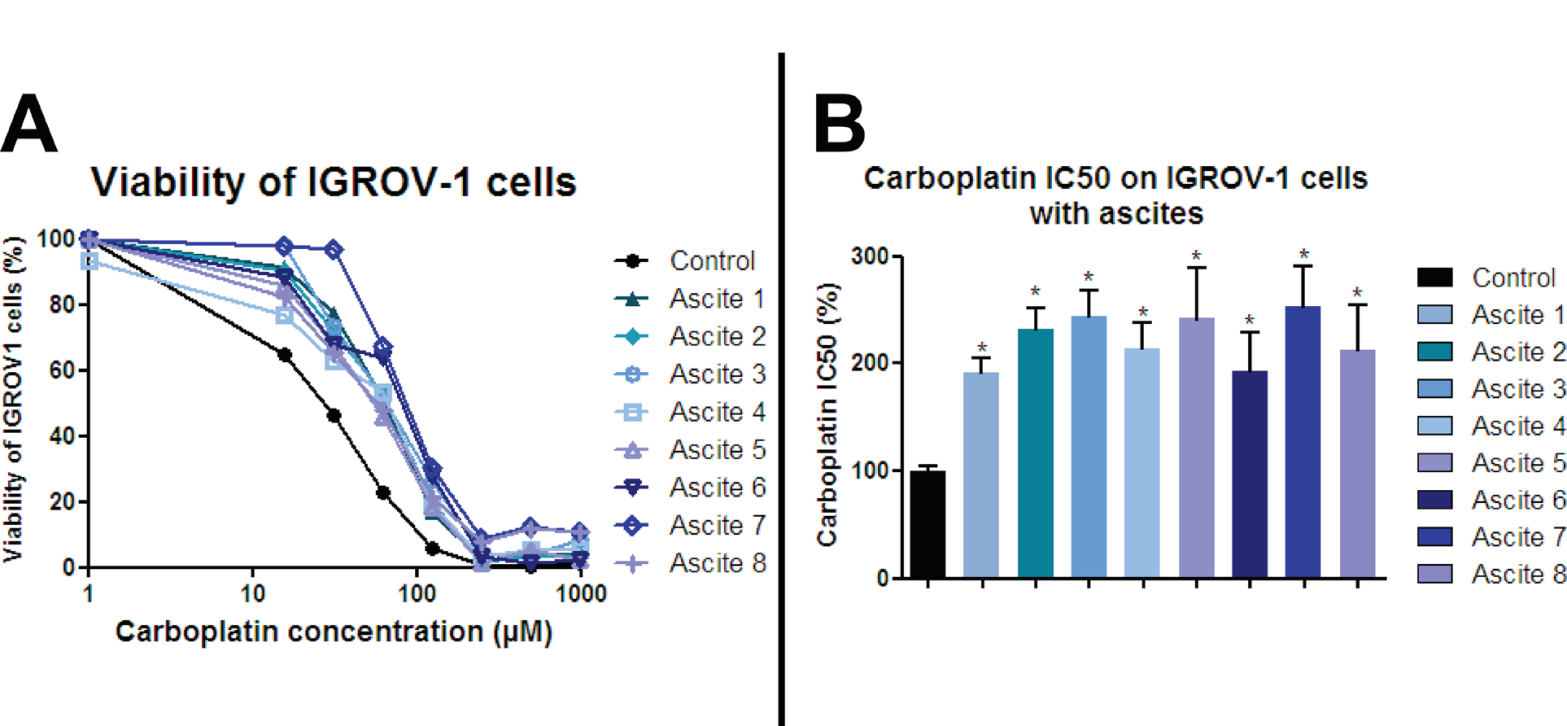
Ascites protect ovarian cancer cells from carboplatin-induced growth inhibition HOCCs alone or in the presence of eight different ascites samples were treated with increasing concentrations of carboplatin for 48 h. (**A**) Cell viability was reported for IGROV-1 cells. The IC50 correspond to 50% viability. (**B**) IC50 values are reported in the histograms below. Results correspond to mean ± SEM of triplicates. Data are representative of three independent experiments.

Platinum-based chemotherapy induces ovarian cancer cell growth inhibition and apoptotic death and is used in the management of ovarian cancer patients. We analyzed whether carboplatin-mediated growth inhibition of the ovarian cancer cell line IGROV-1 could be impaired by secreted factors in ascites from patients who relapsed after a first chemotherapy treatment. First, we determined the carboplatin concentration needed to inhibit 50% IGROV-1 cell growth (inhibitory concentration 50, IC50). IGROV-1 cells, a prototype of high-grade serous carcinoma, were chemosensitive to carboplatin treatment with a dose-dependent decrease in cell viability. The IC50 of the carboplatin on the IGROV-1 cells grown in the control medium was used as a reference (100%). When the IGROV-1 cells were mixed with and cultured in medium containing 50% ascites, we observed an increase in their chemoresistance as shown by an increase in IC50 ranging from 192 to 253% (Figure 1) depending on the patient sample. Whether these ascites came from patients with serous (1–9, 11) or clear cell carcinoma (10), from those with low grade (1) or high grade cancer (10–11), or from those who were sensitive or refractory at the beginning of the treatment, made no significant difference (Table 1 and Figure 1). These results indicate that ascites from most patients contained secreted factors which could enhance chemotherapy resistance of IGROV-1 cells. The fact that secreted factors present in ascites could induce chemoresistance acquisition was also observed using another ovarian adenocarcinoma cell line (JHOC-5), a prototype of clear cell carcinoma.

**Table 1:** Clinical data of the 8 patients with ovarian cancer from whom the ascites samples were taken, including pathology, treatment strategy, disease extension at surgical procedure measured by the PCI (Peritoneal Cancer Index), response to chemotherapy and survival

Patient number	Age	Cancer	Type	Grade	Stade	PCI	Number affected regions	Ascite volume (l)	Chemotherapies before collection	Chemotherapies after collection	Sensitivity	Relapse	Death
1	76	Ovarian adenocarcinoma	Serous	Low- grade	IIIc	9	5	1.2	No	Carboplatin + Paclitaxel	Sensitive	No recurrence even after 5 years	No
2	77	Ovarian adenocarcinoma	Serous	High- grade	III	34	13	5.4	No	Carboplatin monotherapy	Refractory	During treatment	Yes
3	80	Ovarian adenocarcinoma	Serous	High- grade	IV	ND	ND	ND	No	Carboplatin + Paclitaxel	Refractory	During treatment	Yes
4	66	Ovarian adenocarcinoma	Serous	High- grade	III	20	10	4	No	Carboplatin + Paclitaxel	Sensitive	48 months	No
5	75	Ovarian adenocarcinoma	Serous	High- grade	III	20	13	5	No	Carboplatin + Paclitaxel	Sensitive	20 months	No
6	59	Ovarian adenocarcinoma	Serous	High- grade	III	26	11	0.2	Carboplatin + Paclitaxel	Carboplatin + Paclitaxel	Refractory	During treatment	Yes
7	74	Ovarian adenocarcinoma	Clear cells	ND	IIIc	ND	ND	ND	Carboplatin + Paclitaxel	Carboplatin + Paclitaxel + everolimus	Refractory	During treatment	Yes
8	66	Ovarian adenocarcinoma	Serous	High- grade	III	27	11	5.4	No	Carboplatin + Paclitaxel	Sensitive	7 months	Yes

### Ascites activate both JAK/STAT and PI3K pathways on ovarian cancer cell lines

**Figure 2:**
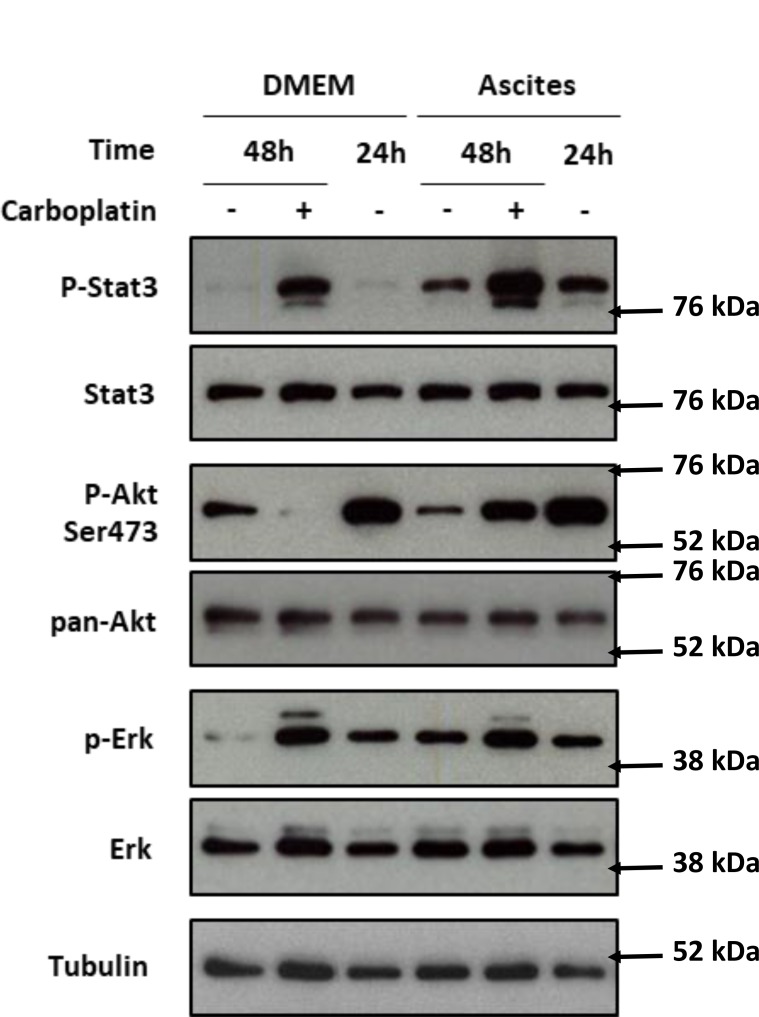
PI3K/Akt, STAT3 and MAPK protein pathway activation after IGROV-1 incubation with ascites and treatment with carboplatin IGROV-1 cells were exposed to DMEM alone (DMEM) or mixed with 50% ascites (Ascites) for the indicated time. Twenty four hours after cell seeding (recorded 24 h), they were treated with (+) or without (–) carboplatin for 24 h more (recorded 48 h). Akt, Erk and Stat3 level and their activating phosphorylation were analyzed by western blot. The protein expression was normalized to tubulin expression. The expression levels of the phosphorylated forms of Akt, Erk and Stat 3 were normalized to that of their corresponding total protein.

To study the effect of ascites in HOCCs, we chose to focus on three signaling pathways potentially implicated in the acquisition of carboplatin resistance. We cultured IGROV-1 in medium containing 50% ascites in the presence or absence of carboplatin (50 µM) and extracted cell proteins at different time points (0, 24 h, and 48 h) after ascites and carboplatin exposition. We evaluated JAK/STAT, MAPK and PI3K/Akt activation through protein phosphorylation (Figure 2). A 48 h exposure to carboplatin induced STAT3 phosphorylation in the presence or absence of ascites. The incubation of cells in the presence of ascites alone for 24 h was also able to induce a STAT 3 phosphorylation that was not increased at 48 h. We observed a slight additive effect of the carboplatin exposure and ascites stimulation (Figure 2). Taken together, our results suggest that the STAT3 pathway could be involved in chemoresistance mediated through the release of factors into the ascites The 48 h carboplatin exposure resulted in a decrease in Akt phosphorylation that was prevented specifically on serin 473 (S473) in the presence of ascites. Without carboplatin, we found an increase of Akt S473 phosphorylation as well as Erk phosphorylation in IGROV-1 cells cultured with patient ascites (Figure 2). Carboplatin did not interfere with the Erk phosphorylation induced by ascites. These data indicate a potential implication of both PI3K/Akt and STAT3 pathways in ovarian cancer cell acquisition of chemoresistance likely through factor release into the ascites.

### Correlation between IL-6 concentration and ascites-induced chemoresistance

**Figure 3:**
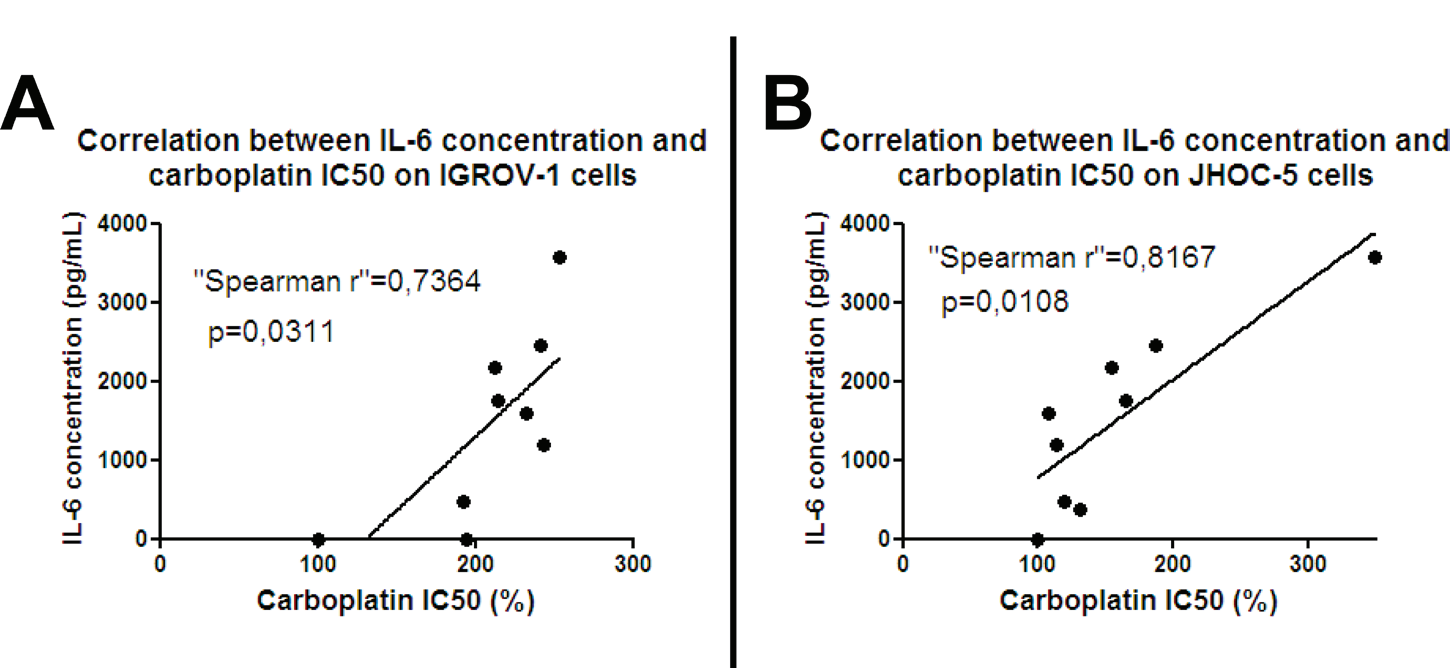
Correlation between IL-6 and chemoresistance: IGROV-1 cells (**A**) or JHOC-5 cells (**B**) were exposed to eight patient-derived ascites samples containing different concentrations of IL-6. Cells were treated with different concentrations of carboplatin 12 hours later, then after another 48 hours the cell viability was evaluated. IC50 of carboplatin was determined and correlated with the amount of IL-6. Spearman rank correlation was used to test the correlation between IC50 and IL-6 concentration in ascites.

Ascites have been found to contain several factors, including chemokines, with potential involvement in chemoresistance [31–34]. Among them, we focused on the cytokine IL-6 which has already been implicated in chemoresistance and angiogenesis in several malignancies including ovarian cancer [35]. We measured IL-6 concentration in ascites by ELISA and correlated it with patient prognosis. In concordance with reported data [36], high levels of IL-6 in ascites correlated with poor prognostic outcome. Different ascites from eight patients induced different levels of chemoresistance (Figure 1). IL-6 concentration positively correlated with the IC50 of carboplatin for both IGROV-1 cells (Figure 3A) and the JHOC-5 cell line (Figure 3B).

### Effect of the IL-6/STAT3 pathway inhibition in HOCC chemoresistance induced by ascites

**Figure 4:**
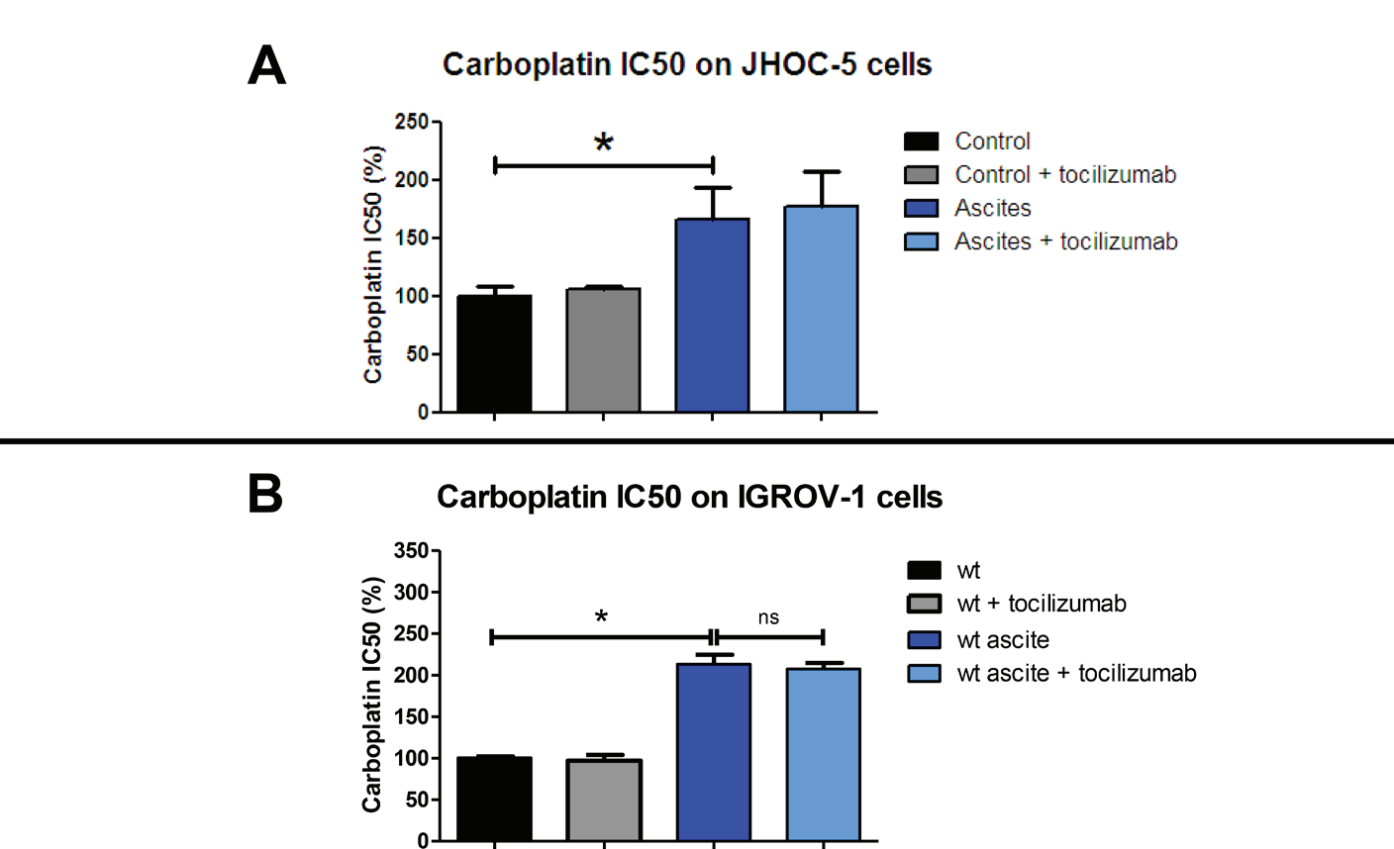
Effect of tocilizumab treatment on ascites in the chemoresistance acquisition of two HOCC lines: JHOC-5 (**A**) and IGROV-1 (**B**). Cells were treated with 8 different ascites samples combined or not with tocilizumab (1 µM) for 12 hours. Carboplatin was then added and cell viability was evaluated 48 h later. Results correspond to mean ± SEM of triplicates of experiments performed using 8 different ascites samples.

We decided to treat IGROV-1 and JHOC-5 cell lines exposed to ascites with an antibody directed against IL-6 in the hope of reversing the chemoresistance. To this end, we added tocilizumab, an antibody directed against IL-6 already used in the treatment of polyarthritis, to cell culture medium alone or in combination with ascites. After 12 hours culture, we then added different concentrations of carboplatin before evaluating cell viability 48 hours later. The JHOC-5 clear cell carcinoma cell line bears the tumor suppressor gene *ARID1A* (encoding BAF250) and *PIK3CA* mutations, which cooperate to promote tumor growth through IL-6 overproduction [37, 20]. We used the eight different ascites reported in Figure 1. As was observed for IGROV-1 cells, all ascites induced different levels of chemoresistance acquisition in the JHOC-5 cells. Tocilizumab did not reverse the chemoresistance in either cell line (Figure 4A: JHOC-5 cells and Figure 4B: IGROV-1 cells). Indeed, the mean IC50 of carboplatin observed in the presence of these 8 ascites was not modified despite treatment with tocilizumab.

### Expression of class I PI3K isoforms in HOCCs

**Figure 5:**
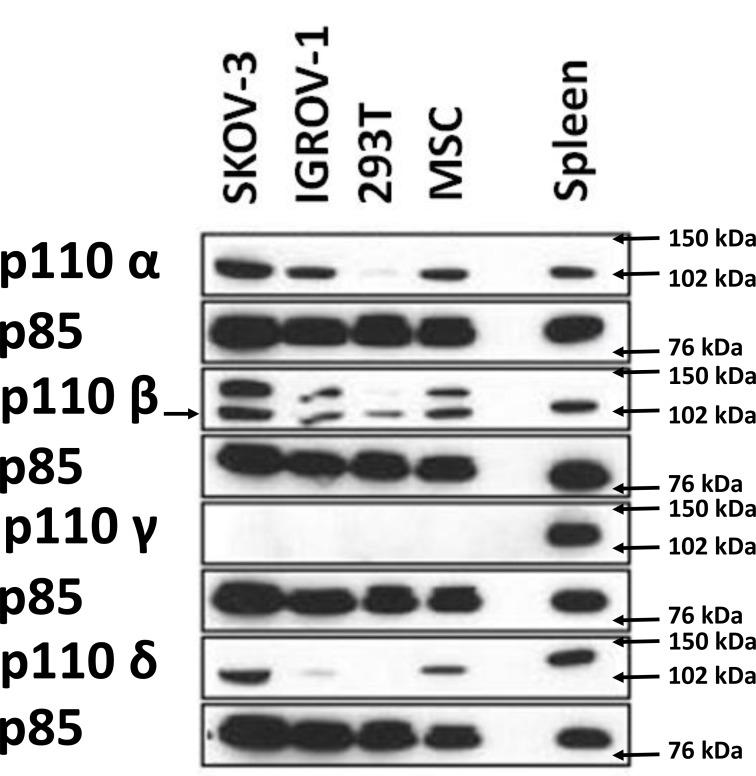
Western blot analysis demonstrating the expression of different class I PI3K isoforms on 2 HOCC lines as well as on mesenchymal stem cells from the tumor microenvironment Human embryonic kidney cells (HEK 293) were used as negative control, while immune cells of splenic tissue known to express high levels of p110δ and p110γ were used as positive control for expression levels of these isoforms. The expression of PI3K isoforms was normalized to p85 expression.

**Figure 6:**
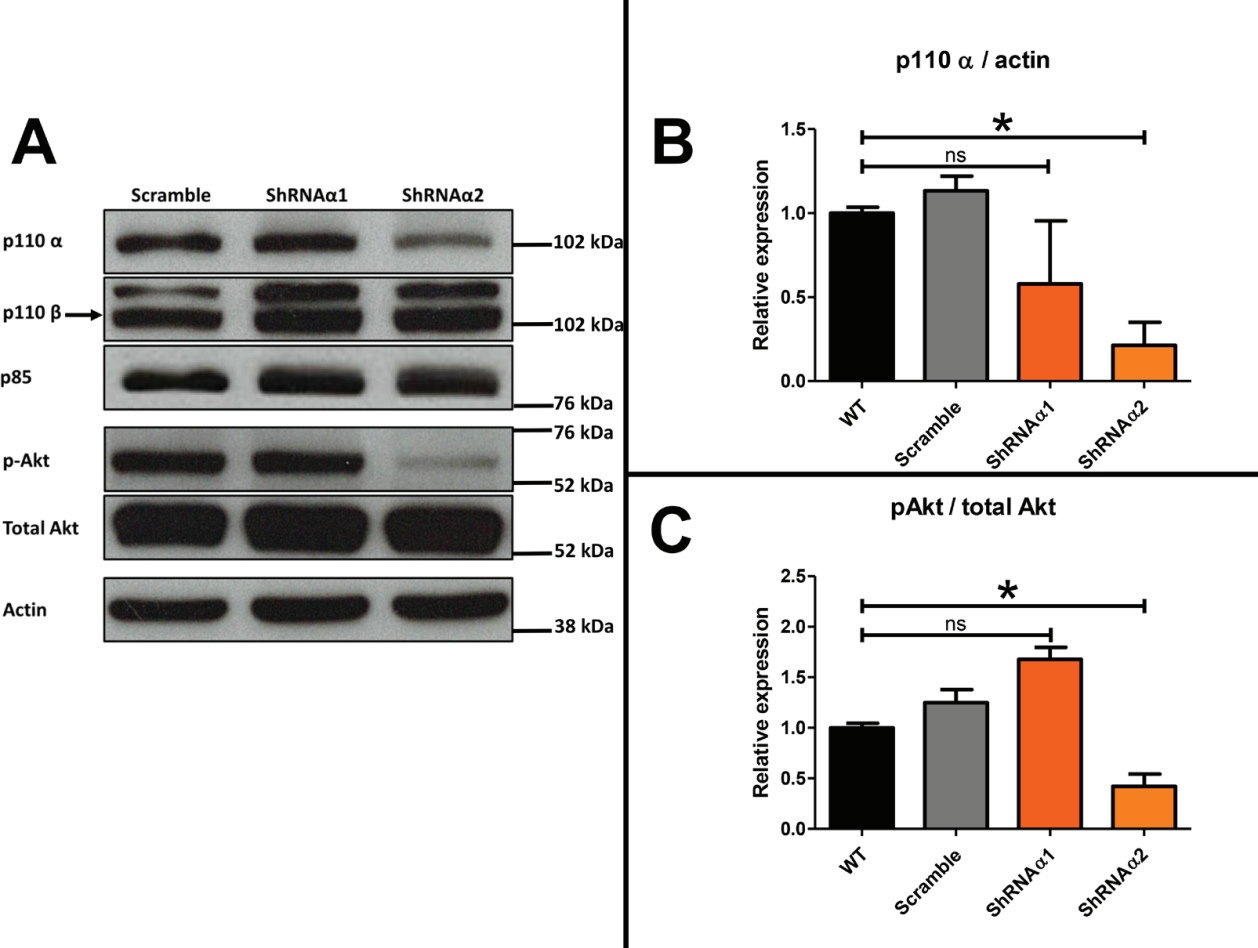
(**A**) Western blot analysis showing the level of class I PI3K isoforms in ovarian cancer cells transduced with scramble shRNA or shRNA directed against PI3KCA (α1 and α2) and Western blot analysis demonstrating the effect of this inhibition, mediated by the two vectors shRNAα1 or shRNAα2, on the phosphorylation of S473 Akt. The expression of PI3K isoforms and S473A Akt were normalized to p85 expression. (**B**) Results were represented in fold change of p110α compared to the respective untreated control. (**C**) Results were represented in fold change of pAkt compared to the respective untreated control.

Addition of an anti-IL-6 antibody thus appeared insufficient to reverse the chemoresistance acquisition mediated by the ascites. We decided to test whether the combination of JAK/STAT and PI3K/Akt inhibition could abolish chemoresistance of HOCCs. We first checked the expression of the different PI3K isoforms in IGROV-1 as well as in another HOCC line (SKOV-3 cells), chosen as both are mutated for *PI3KCA*, encoding the catalytic subunit p110α of PI3K. As shown in Figure 5, both cell lines expressed p110α and p110β, and to a lesser extent p110δ, though no p110γ. We also checked PI3K isoform expression in mesenchymal stem cells (MSCs) representing the tumor microenvironment. Indeed, these cells could be involved in IL-6 secretion into the ascites, as described by Castells *et al.* [38]. The MSCs showed the same pattern of expression of class I PI3K isoforms as the HOCCs. Since PI3KCA polymorphism has been observed in ovarian cancer and because this isoform is implicated in oncogenesis we decided to specifically inhibit this isoform. To evaluate the effect of PI3K pathway blockade we used RNA interference on the HOCCs in both *in vitro* and *in vivo* experiments. We transduced the HOCCs with lentiviral vectors encoding either a scramble shRNA or shRNAs targeting various locations of the *PI3KCA* transcript, and checked the knockdown efficiency by western blot using different shRNA sequences (alpha 1 to alpha 4, alpha 3 and 4 not shown). The scramble shRNA had no effect on the expression of the PI3K isoforms α and β. The shRNAα2 was the most efficient at knocking down *PI3KCA* expression. In addition, unlike shRNAα1, shRNAα3 and shRNAα4 the shRNAα2 effectively reduced downstream Akt signaling, as shown by a decrease in phosphoryled-Akt on S473 (p-Akt S473). We observed no compensation provided by an overexpression of p110β following the abolition of *PI3KCA* expression by shRNAα2. Inhibition of *PI3KCA* alone was able to downregulate the Akt pathway in IGROV-1 cells. We selected the shRNAα2 for following experiments (Figure 6).

### Inhibition of p110α did not decrease IGROV-1 chemoresistance induced by ascites

**Figure 7:**
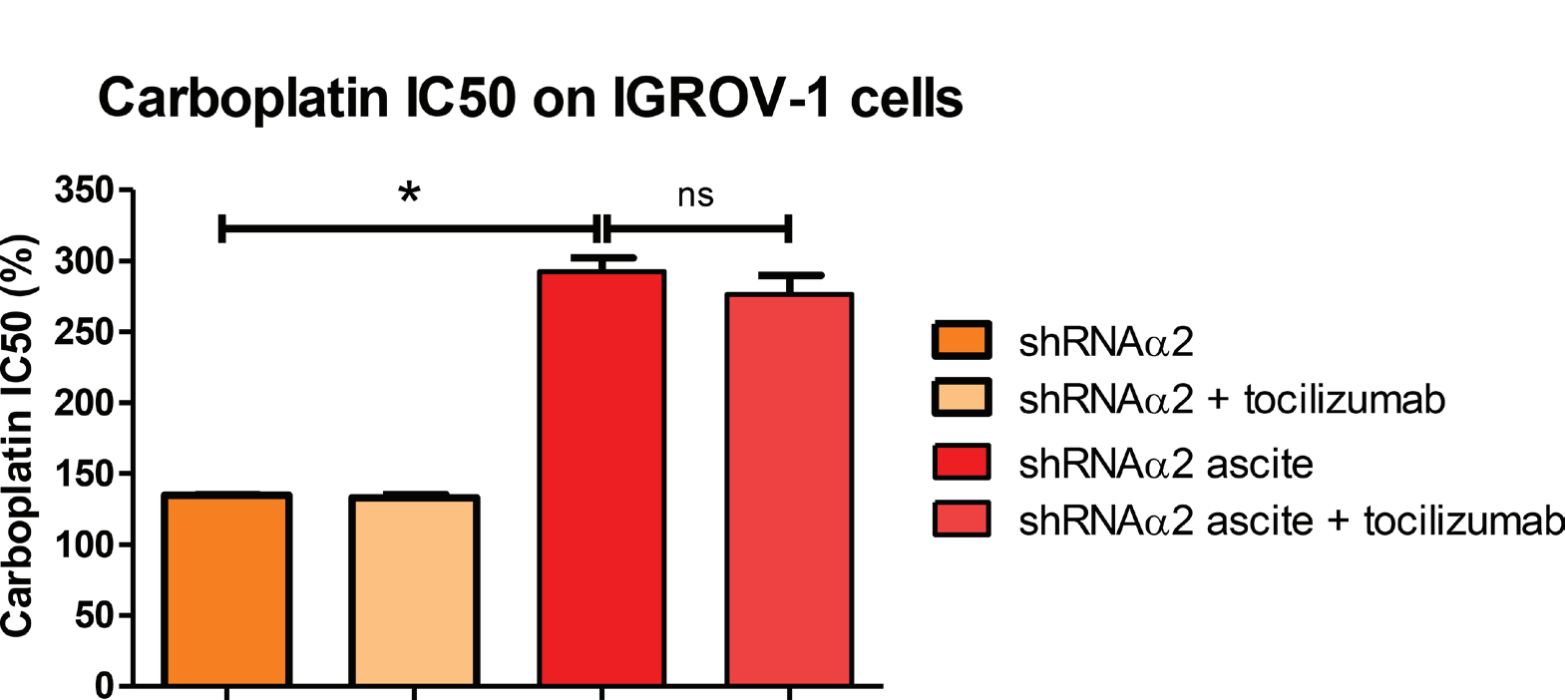
Ascites protect HOCCs from carboplatin-induced growth inhibition, even in the presence of tocilizumab PI3KCA inhibited IGROV-1 cells cultured alone or in the presence of different ascites in addition to tocilizumab (1 µM) were treated with increasing concentrations of carboplatin for 48 h and are compared to IGROV-1 cells WT (corresponding to 100%). Cell viability (mean IC50 values) was reported for the IGROV-1 cells. The results correspond to mean ± SEM of triplicates. Data are representative of two independent experiments.

As demonstrated above, incubation of the IGROV-1 WT during 24 h in the presence of ascites promoted an increase in IC50 dose of carboplatin. We postulated upon the involvement of Akt phosphorylation on S473 in this phenomenon and hypothesized that ascites secretions may activate the anti-apoptotic signaling pathway through p110α activation. Incubation with the ascites conferred an increase in resistance to carboplatin whether the cells expressed or not high levels of PI3KCA. The acquisition of chemoresistance was therefore not reversed by an inhibition in PI3KCA expression (Figure 7).

### Combined inhibition of IL-6/STAT3 pathway and p110α does not reverse IGROV-1 chemoresistance induced by ascites

We next investigated whether combinatorial blocking of IL-6/STAT3 and PI3K/Akt pathways would have an effect on tumor growth and response to chemotherapy. Blockade of both IL-6 and PI3KCA had no synergistic effect and failed to reverse chemoresistance acquisition through secreted factors in the ascites. Surprisingly, inhibition of the isoform p110α in fact increased resistance to carboplatin in the IGROV-1 cell line (Figure 7).

Taken together, our results show that treatment with an antibody against IL-6 did not reverse chemoresistance to carboplatin, even upon PI3KCA inhibition.

## DISCUSSION

Ascites microenvironment secreted factors can induce platinum chemoresistance in ovarian cancer. One of the potential mechanisms of chemoresistance occurs through activation of the STAT3/PI3K/Akt axis, as demonstrated by increased STAT3 and Akt phosphorylation (Figure 2). This Akt phosphorylation inducedanti-apoptotic signaling, thereby increasing cell survival. Phosphorylation of Akt increased upon exposure to ascites medium (Figure 2), suggesting a PI3K/Akt activation induced by secreted factors present within.

Literature data demonstrate that IL-6 is one of the major immunoregulatory cytokines present in the tumor microenvironment and found overexpressed in almost all types of tumor. It is produced by immune cells, MSCs and tumor cells [29] and has a number of tumor-promoting activities including its capacity to induce tumor proliferation, invasion, angiogenesis and chemoresistance [39, 40]. Not surprisingly therefore, it is associated with poor prognosis in patients. IL-6 stimulates tumor cell proliferation and survival by activating the PI3K/AkT and JAK/STAT pathways *via* gp130 tyrosine phosphorylation [35]. It regulates the process of apoptosis by activating STAT-3 and NF-kB signaling, alongside the transcription of anti-apoptotic genes coding for Bcl-2, Bcl-xL, and Mcl-1. IL-6 also supports tumor cell survival by inducing the expression of survivin through direct binding of STAT-3 to the survivin promoter. It is also implicated in the development of epithelial ovarian cancer induced ascites [30] and can be found at high levels in ascites and serum. Expression levels are upregulated in ovarian cancer samples [35, 41, 42] in which correlations have been found with tumor burden, optimal cytoreduction, ascites and survival in some studies [42–44], though controversially not in others [45–47].

IL-6 can induce resistance towards anticancer therapy induced death pathways. Indeed, it has been shown to confer protection from dexamethasone-induced apoptosis by activating PI3K/Akt signaling and inactivating caspase-9, thereby inhibiting apoptosis in multiple myeloma cells [35]. IL-6/STAT3 is the main pathway through which IL-6 regulates tumor-promoting activities. Our results indicate that the activation of STAT3 was mediated by secreted factors present in the ascites. Therefore, we sought to block the STAT3 signaling pathway independently or in combination with conventional chemotherapy as a potential treatment strategy in tumors showing high levels of IL-6 in the tumor microenvironment [35]. Our results using tocilizumab, an IL-6R blocking antibody, failed to demonstrate any reversion of chemoresistance to carboplatin acquired by either IGROV-1 or JHOC5 cells cultured in the presence of ascites from patients. These results are concordant with other studies demonstrating the poor effect of IL-6 inhibition on cell survival *in vitro*. Although IL-6 inhibition reduces the constitutive production of inflammatory mediators, it has no impact on tumor growth or cell survival because of the lack of stromal reaction [47]. Indeed, results from *in vivo* models show that neutralizing IL-6 enhances the therapeutic effect of paclitaxel in an ovarian cancer mouse model, leading to reduced tumor growth and angiogenesis [47]. However, a phase I/II trial of siltuximab (anti-IL-6 monoclonal antibody) in ovarian cancer patients demonstrated a lack of clinical efficacy [48], arguing that IL-6 inhibition could eliminate IL-6 sensitive clones leading to the development of resistant clones whose growth is triggered by other pathways such as the gp130 family [49]. Indeed, serum concentrations of the gp130 subunit of the IL-6R were unaffected by siltuximab treatment in patients with ovarian cancer [48]. The subunit gp130 activates cytoplasmic tyrosine kinases, resulting in the phosphorylation of downstream transcription factors [35]. In this way, IL-6 could trigger the PI3K/Akt, NF-κB and MAPK/ERK signaling pathways in a manner similar to that observed in other tumors such as prostate cancer with the upregulation of cyclin A1 [50], or bladder cancer and multiple myeloma [35, 21]. Thus, secreted IL-6- could act in an autocrine manner on ovarian cancer cell surface receptors [49] as well as activating the PI3-kinases.

Various genetic alterations that induce increased PI3K/Akt/mTOR signaling have been found in ovarian cancer. Somatic activating mutations in the *PIK3CA* gene were found in more than 10% of high-grade serous and 40% of clear cell ovarian carcinomas [50], and constitutive activation of the PI3K/Akt/mTOR axis is known to confer drug resistance to many types of cancer. Therefore, we decided to investigate the protein expression of the different PI3K isoforms in ovarian cancer. The HOCCs expressed high levels of the PI3K isoforms α and β and their acquisition of chemoresistance through culturing with ascites was associated with the activation of PI3K/Akt and IL-6/STAT3 pathways. We next focused on the implication of the PI3K catalytic subunit p110α in platinum resistance, and on the combined inhibition of IL-6/STAT3 and PI3K/Akt pathways to revert chemotherapy resistance. One of the major interests in using isoform-selective PI3K inhibitors is the reduced toxicity in comparison with the use of pan-PI3K inhibitors, in particular in combination therapies. We used shRNA instead of PI3KCA pharmacologically specific inhibitors to better mimic the inactivation of the *PI3KCA* gene in tumor cells, thus avoiding p110α inhibition in stromal cells. Knockdown of p110α expression alone or in combination with IL-6/STAT3 inhibition did not reduce ovarian cancer cell resistance to carboplatin induced by ascites medium. This could be explained by the loss of negative feedback loops normally induced when the PI3K/Akt/mTor pathway is active [50]. Our data show that p110β expression was not affected by p110α knockdown; we did not evaluate mTOR1 and 2 expressions and activity. The effect of p110α inhibition was previously associated with increased activity of another PI3K isoform, such as p110β, without change in protein expression [51]. This cancer cell response to signal-targeted therapies is referred to as adaptive response. As suggested by other authors, it is necessary to combine PI3K/Akt/mTOR inhibitors with other agents to enhance their potential [52]. Other ovarian cancer cell types might benefit more from targeting IL 6 and PI3K pathways, such as clear cell HOCCs (JHOC5). In one murine model of clear cell carcinoma of the ovary, *PIK3CA* mutations drove signaling loops to promote high levels of IL-6 production in the absence of negative regulation by ARID1A [53, 42]. Another limitation of our study is the absence of co-culture with different stromal components, which shape intracellullar signaling networks and possibly modify the sensitivity to drug combinations. Indeed, as has been shown in leukemia models, PI3K chemoresistance could, at least in part, be mediated by other cells of the tumor microenvironment. Bone marrow stroma has been shown to protect acute myeloid leukemia cells from inhibition by an anti-CD44 antibody in part via PI3K/Akt signaling [54]. Inhibition of p110α blocked bone marrow stromal cell-derived migration and survival and reduced drug resistance of chronic lymphocytic leukemia [28]. In another model of osteosarcoma, mesenchymal stem cell media promoted chemoresistance via IL6 secretion [55].

IL-6/STAT3 and PIK3CA inhibition did not reverse platinum chemoresistance in ovarian cancer and IL-6 levels cannot be considered as a marker of sensitivity to platinum-based chemotherapy. This dual inhibition could however be further explored in other ovarian cancer models including stromal microenvironment.

## MATERIALS AND METHODS

### Cell culture

Human ovarian cancer cells (HOCCs) IGROV-1, SKOV-3 and JHOC-5 were obtained respectively from the institute Gustave Roussy [18], the American Type Culture Collection (Manassas, VA, USA) (HTB-77) and the RIKEN institute (Japan) (RCB 1520). Mesenchymal stem cells (MSCs) were obtained from the EFS Institute, Toulouse, France. HOCCs and MSCs were cultured respectively in RPMI or DMEM medium supplemented with 10% fetal calf serum, penicillin/streptomycin (100 UI/ml/100 mg/ml) and 2 mM L-Glutamine (Cambrex Biosciences, Milan, Italy). Cell lines were routinely checked for mycoplasma.

### Lentiviral vector

LVTHM^®^ is a lentiviral vector (LV) encoding shRNA under the control of the H1 promoter [17] as well as GFP under the control of the EF1 promoter. Four different lentiviral vectors were constructed corresponding to various targeted regions of *PI3KCA*: Alpha 1: 5′ CGCGTCCCCGCGAAATTCTCACACTATTATTTCAAGAGAATAATAGTGTGAGAATTTCGCTTTTTGGAAAT 3’, Alpha 2: 5′ CGCGTCCCCGCACAATCCATGAACAGCATTTTCAAGAGAAATGCTGTTCATGGATTGTGCTTTTTGGAAAT 3′; Alpha 3: 5′ AATTCGCATTAGAATTTACAGCAAGATCTCTTGAATCTTGCTGTAAATTCTAATGCC 3′ Alpha 4 TCGAGCCAGATGTATTGCTTGGTAAATTCAAGAGATTTACCAAGCAATACATCTGGG 3′.

The same lentiviral vector encoding a scramble shRNA 5′ (AATTCTTCTAGAGATAGTCTGTACGTTTCAAGAGAACGTACAGACTATCTCTAGAAG) was used as negative control. 293T cells were kindly provided by Genethon (France). Generation of 293T- LVTHM-shalpha 1 to 4 or control and preparation of high titer LV pseudotyped with VSV-G protein have been described previously [18]. 50 × 10^3^ cells were plated on 35 mm dishes 24 h prior to transduction with viral vectors at a MOI of 10:1. Genetically modified cells were sorted by FACS according to GFP expression 96 hours after transduction.

### Conditioned media

Ascites were collected from patients during exploratory laparoscopy for peritoneal carcinomatosis of ovarian origin. Only ascites from tumors with adenocarcinoma histology were selected. The description of the disease stages is presented in Table 1. All patients had previously given their informed consent. Ascites were centrifuged for 10 min at 400 g and the supernatant was filtered at 0.2 μm.

### IC50 assays

HOCCs were seeded in 96-well plates (5000 cells per well) in the presence or absence of ascites and treated 24 h after with the indicated concentrations of carboplatin between 15.625 and 1000 µM. After 48 h, cell viability was measured using WST-1 colorimetric assay (Roche). Untreated cells were used as reference of 100% viability. To inhibit IL-6 activity, HOCCs were treated with 1 µM tocilizumab (Roche) in combination carboplatin at different concentrations.

### Western-blotting analysis

20 micrograms of protein were separated by SDS-PAGE on a 10% polyacrylamide gel. Proteins were transferred onto nitrocellulose. Membranes were saturated for 1 h in TBS (50 mM Tris, 150 mM NaCl) / 0.1% Tween 20/ 5% milk or 5% bovine serum albumin (BSA) for phosphorylated protein analysis and incubated overnight at 4°C with a rabbit polyclonal primary antibody directed against p85 (1:1000, Millipore clone 06-195), p110α (1:1000, Cell Signaling 4249), p110β (1:1000, Santacruz SC-602), p-AktS473 (1:1000, Cell Signaling #4060S); pan-Akt (1:1000, Cell Signaling #4691S); p-Erk (1:1000, Cell Signaling #4377S), p-Stat3 (1:1000, Cell Signaling #9145) or β-Tubulin (1:1000, Cell Signaling # 2146S); or a mouse polyclonal primary antibody directed against Erk (1:1000, mouse, Cell Signaling #4696) or Stat3 (1:1000, Mouse, Cell Signaling #9139). Membranes were washed three times with TBS/0.1% Tween 20 (TT) then incubated 1.5 h with a secondary antibody (1:10000 anti-rabbit or 1:5000 anti-mouse Cell Signaling Technology) coupled with horse radish peroxidase. Membranes were washed three times with TT. Immunocomplexes were revealed by enhanced chemiluminescence (Pierce).

### ELISA (Enzyme-like immunosorbent assay)

IL-6 was quantified using a specific ELISA (DuoSet^®^ ELISA Development System (R&D Systems) according to the manufacturer’s protocol.

### Statistical analysis

Results are expressed as the mean ± s.e.m. Wilcoxon-Mann-Whitney test was used to compare values of test and control samples in IC50 assays. ^*^p < 0.05 and ^**^p < 0.01 indicate a significant difference. The Spearman rank correlation was used to test the correlation between IC50 and IL-6 concentration in ascites.
